# Performance of component resolved microarray diagnosis in fruits allergy

**DOI:** 10.1186/2045-7022-5-S3-P32

**Published:** 2015-03-30

**Authors:** María José Goikoetxea, Carmen D'Amelio, Rubén Martínez, Francisa Gómez, Eloína González, Francisco Feo-Brito, Carmen Moya, Soledad Terrados, Javier Fernández, Antonio Parra, María Luisa Sanz

**Affiliations:** 1 Clinica Universidad de Navarra, Pamplona, Spain; 2 Hospital Carlos Haya, Malaga, Spain; 3 Hospital Fuenlabrada, Madrid, Spain; 4 Complejo Hospitalario Ciudad Real, Ciudad Real, Spain; 5 Hospital Almeria, Almeria, Spain; 6 Hospital Ramon y Cajal, Madrid, Spain; 7 Hospital de Alicante, Alicante, Spain; 8 Hospital de A Coruña, La Coruña, Spain

## 

Performance of commercially available component resolved microarray has not been widely evaluated in food allergy. Fruit allergy is a prevalent food allergy in which component resolved diagnosis is crucial.

In the context of a multicentric study on pollen and vegetable food allergy in Spain, we enrolled patients (n=137) who had suffered at least two episodes of immediate symptoms after ingestion of fruits (peach, apple or kiwi). Skin prick test was positive for the corresponding nut. ImmunoCAP ISAC 112 was analyzed in all patients and ImmunoCAP to the available components from the studied sources were analyzed (Pru p 1, Pru p 3, Mal d 1, Act d 1, Act d 2, Act d 5, Act d 8) in those patients showing negative result to ISAC.

Among the 107 patients allergic to peach, 7 were sensitized to Pru p 1 and 90 to Pru p 3. Fifteen peach-allergic patients showed no sensitization to any of these components in the microarray. Among these 15 ISAC negative peach allergic patients, 2 were sensitized to Pru p 1 and 9 to Pru p 3 by CAP.

Among the 25 patients allergic to kiwi, 6 were sensitized to Act d 1, 3 to Act d 2, 1 to Act d 5 and none to Act d 8. Sixteen patients allergic to kiw showed no sensitization to any components in the microarray. Among these 16 ISAC negative kiwi allergic patients, no patients were sensitized to the only available kiwi component by CAP Act d 8.

Among the 52 patients allergic to apple, 6 were sensitized to Mal d 1. Forty-six apple allergic patients showed no sensitization to any components by the microarray technique. Forty-two were sensitized to Pru p 3 by ISAC. Among the 46 ISAC negative apple allergic patients, none were sensitized to Mal d 1 by CAP.

Among the 137 patients allergic to peach, apple or kiwi, 67 patients (49%) did not show specific IgE against the studied nuts/ components in ISAC.

Sensitization to the lipid transfer protein Pru p 3 not detected by ISAC was recovered by CAP analysis in 60% of the cases. In apple allergic patients LTP sensitization is very prevalent in our area. Therefore, Mal d 3 should be included in a new version of ISAC.

